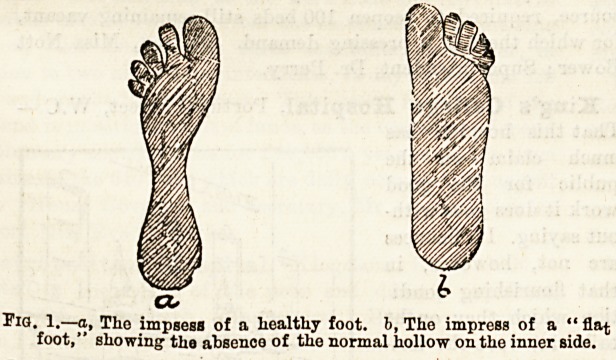# Nature and Treatment of Flat Foot

**Published:** 1893-12-23

**Authors:** Walter L. Woollcombe

**Affiliations:** Assistant Surgeon South Devon and East Cornwall Hospital, Plymouth


### THE HOSPITAL CLINIC.

### NATURE AND TREATMENT OF FLAT-FOOT.

By WALTER L. WOOLLCOMBE, F.R.C.S., Edin., Assistant Surgeon South Devon and East Cornwall Hospital, Plymouth.

In this article I wish to call attention to a class of cases with which every practitioner, more especially those connected with the staff of a hospital, must come into almost daily contact, and which occur in people to whom the helplessness produced by the deformity is such a serious consideration, that it is important we should be agreed upon some simple and rapid plan of treatment.

Flat-foot, or *talipes valgus*, as it is, I think, somewhat badly named, differs from other forms of talipes in that it is due entirely to a relaxed condition of the tarsal ligaments and a flabbiness of certain muscles, and not to any error of development, and hence we meet with it at an age when the rapidly increasing weight of the body makes the greatest demand on the structures which support it, and which are not yet in a fully developed condition to do so, namely, at or about puberty.

For this reason the sufferers are usually errand boys, servant girls, shop assistants, railway porters, telegraph boys, in other words, those who are on their feet for long hours, and probably carrying weights when tired; these are the conditions which, when combined with an insufficient or unsuitable diet, are most fruitful in producing flat-foot, though perhaps one should mention bad shoeing as a frequent accompaniment if not an active factor in producing the condition.

The most prominent symptom is almost without exception pain across the instep generally together with aching in the calf, and on examining the foot one is at once struck by its apparent increased length and breadth, even when the patient is not standing, although it is in the latter position that the deformity is most marked.

A very good gauge of the amount of flattening is obtained by making the patient dip the sole of his foot in a basin of water, and having shaken off the loose drops, place it firmly with his weight on it on some

FIG. 1.—*a*, The impsess of a healthy foot. *b*, The impress of a "flat foot," showing the absence of the normal hollow on the inner side.

flat surface which will show the wet mark. One thus obtains a very good idea of the extent of the inner border of the foot which touches the ground, and by keeping a drawing of the impression one is furnished with a guide from time to time as to the effect of treatment.

The ligamentous structures, whose yielding is chiefly responsible for the deformity, are the plantar fasciæ and the inf. calcaneo-scaphoid ligt., and the most important muscles are the peroneus longus and tibialis posticus. The yielding of these from the causes mentioned above, or from a sprain (which should have been mentioned), allows the head of the astragalus to fall downwards and inwards, pushing with it the scaphoid, whose tubercle forms a striking prominence, as does the head of the astragalus in an advanced case.

This tilting over of the ankle, and the alteration in the direction in which the weight of the body is transferred to the ground necessarily tips up the outer border of the foot, and it is to this cause and not to any active contraction of structures on the outer side of the foot that the valgus is due; hence any division of structures here, such as the tendon of the peroneus langus which has been advised, is, in my opinion, entirely opposed to the object at which we should aim, and in the attainment of which the above-mentioned muscle and tendon is our chief helper.

With regard to the details of treatment, it is best, I think, to make a division into two classes: (a) Those in which the muscles are not strong enough to support the arch, but the ligaments have not given way to any great extent so that there is little bony deformity. (b) Those in which the ligaments have become much stretched so that the scaphoid and head of astragalus form well-marked projections on the inner border of the foot.

The treatment of the former, provided the patient can give up for a time the occupation which has produced the deformity, consists in daily massage of the calf and foot, with a series of exercises involving rising on the toes, at first without additional weights, but after a time with a gradually increasing weight in the hands, at the same time a rational diet, and tonics such as iron and strychnia.

The latter condition is the one for which we are usually consulted, as it is not until there is considerable deformity and much pain from tarsal synonitis and adhesions that patients seek relief, and it is just in these cases that we can do so much to make them useful members of the community.

It is best at once, I think, to give an anæsthetic and thoroughly break down all adhesions between the tarsal bones, especially the astragalus scaphoid and cuneiforms. After this it will be found that the arch of the foot can be restored to a great extent, and this having been done, the foot should be put up in plaster with a pad under the astragalus.

Personally, I use a rubber pad shaped like a quarter of a sphere, the rounded convex part fitting into the arch of the foot. The plaster case should be kept on about three weeks, and when taken off systematic douching and massage should be carried out for another week before any weight is allowed to be borne on the foot, which should meanwhile be fitted with a good boot straight along the inner edge, and blocked high at the toes, and into this boot should be fitted one of the above described rubber pads, the two flat surfaces resting one against the sole of the boot, and one against the inner side, while the foot rests on the convex surface.

I find by far the best plan is to have the pad sewn between two layers of leather cut out as a sole for the boot. This can be changed from one pair of boots to another, and prevents any slipping of the pad.

Much standing must not be allowed at first, and, above all, not when tired.

Gradually by well devised exercises the calf muscles must be improved in tone, and in cases where patients can and will carry out directions the pads may eventually be left out, but care must be taken that the ligaments have sufficiently consolidated and the tendons supporting the arch acquired sufficient strength to maintain the arch before this is done.

This treatment takes some time and care, but anyone who has witnessed the misery with which the unhappy possessor of a flat-foot goes about his work will not begrudge the time or trouble.

I have made no mention of rheumatism as a factor in producing flat-foot, believing it to be a subsequent condition grafted on to the inflamed and adherent tarsal joints which disappears when the adhesions are broken down and the inflammation subdued by rest.

## Figures and Tables

**Fig. 1. f1:**